# Trajectories of victimization to violence among incarcerated women

**DOI:** 10.1186/s40352-021-00144-8

**Published:** 2021-07-27

**Authors:** Preeta Saxena, Nena Messina

**Affiliations:** 1 Sociology Department, Institutional Research, Planning and Institutional Effectiveness, College of the Canyons, Santa Clarita, CA USA; 2 UCLA Integrated Substance Abuse Programs and Envisioning Justice Solutions, Inc., University of California, Los Angeles, Los Angeles, CA USA

**Keywords:** Violence, Perpetration, Women, Girls, Age, Victimization, Trauma, Incarcerated

## Abstract

**Introduction:**

Limited research has focused on the trajectories of victimization to violence in women’s lives. Furthermore, literature assessing women’s use of violence has primarily focused on adult risk factors (e.g., substance use and criminal histories). Drawing from the pathway’s framework, we explored the impact of multiple forms of childhood victimization and subsequent harmful behaviors on adult-perpetrated violence among women convicted of violent or serious crimes.

**Methods:**

This secondary data analysis included a sample of 1118 incarcerated women from two prisons. Based on prior literature outlining the lifelong negative impact of childhood victimization, we hypothesized that cumulatively, occurrence of abuses, arrest as a minor, number of lifetime arrests, and poly-substance use prior to incarceration, would increase the likelihood of perpetration of multiple forms of violence. GEE regression models were used to examine the relationship between the predictors and adult perpetration of intimidation and physical violence.

**Results:**

Experiences with childhood victimization, early (under age 18) and ongoing criminal justice involvement, and substance use significantly increased the likelihood of adult perpetration of violence, regardless of the type of violence measured (intimidation or physical violence).

**Conclusion:**

Given the documented high prevalence of childhood trauma and abuse among justice-involved women, findings from this study can be used to promote the implementation of trauma-specific treatment for at-risk juvenile girls, whose trajectories of violence might be mitigated.

## Introduction

Justice-involved men have been the primary focus of research on violence in the United States, as they comprise 62% of incarcerated violent offenders nationwide (Bronson & Carson, 2019). However, the remaining 38% of incarcerated violent offenders are women, with sparse research exploring their trajectories of violence. By 2019, the number of incarcerated women in the United States grew over 7 times higher than in 1980, with over 230,000 women in prisons and jails across the country (Carson, 2020), and rose globally by 53% since 2000 (Walmsley, 2017). Critical policy changes and harsher sentencing laws for drug-related crimes played a crucial role in the increase of women’s incarceration; however, recent legislative changes in California (Realignment AB 109, 2014) currently divert supervision of non-violent, non-serious and non-sex, offenders to county jails. The California prison population is now largely composed of men and women convicted of violent crimes (i.e., crimes involving force, threats of force, or use of a weapon, and include offenses such as homicide, manslaughter, assault, and sex offenses), with over 6500 women currently incarcerated. Understanding and responding to the needs of these women, particularly those who have co-occurring disorders, becomes crucial in the prevention of women’s perpetration of violence and in their increased well-being (Saxena et al., 2015).

Existing studies investigating risk factors for women’s use of violence have centered on adult criminal and substance use behaviors with few studies highlighting early childhood experiences, such as abuse and trauma. To develop programs and policies surrounding women’s use of violence, it is crucial to explore the long-term and complex consequences of childhood victimization; the documented correlation with adolescent and adult antisocial behaviors (Grella et al., 2005; Messina & Grella, 2006), and as a risk factor for women’s perpetration of violence and aggression (Kubiak et al., 2017). The high prevalence of adverse childhood experiences (ACEs), lifelong trauma exposure, and substance use disorders among incarcerated women has been widely recognized (Messina & Grella, 2006; Saxena et al., 2016; Tripodi & Pettus-Davis, 2013). In comparison to women in the general population, incarcerated women report a significantly higher prevalence of ACEs (e.g., emotional, physical, sexual abuse, and multiple forms of household dysfunction under the age of 18) and more varied types of trauma (Grella et al., 2013; Fazel et al., 2006; Tusher & Cook, 2010). Also, in comparison with incarcerated men, studies similarly show a higher prevalence of ACEs, a stronger correlation among types of ACEs, continued victimization into adolescence/adulthood, a more pronounced intergenerational impact, and greater severity of chronic mental/physical health outcomes among women (Black et al., 2010; Grella et al., 2005; Harlow, 1999; Kernsmith, 2006; Leban & Gibson, 2020; Messina et al., 2007).

Although childhood trauma and abuse are recognized as experiences which can result in lifelong negative effects, gaps in knowledge regarding childhood victimization and the relationship to the use of violence among women remain. The existing literature has typically focused on women’s engagement in violence against their partners (Magdol et al., 1998). Additional literature regarding women’s use of violence is outlined in following sections.

### Perpetration of violence and intimidation among women

#### Intimate partner violence (IPV)

Power and control have been theorized to be the underlying motivation of men for acts of physical and sexual violence against women; yet stalking, aggressive, threatening, and intimidating behaviors are also serious forms of abuse and control (Shorey et al., 2008). Some studies posit that women can also express hostility and aggression through psychological efforts to demean and intimidate their partners as an effective method of control without violence (Swan & Snow, 2003). As part of a large literature review on IPV, Langhinrichsen-Rohling et al. (2012) conducted a review of 18 studies comparing men and women’s reported motivation for IPV (i.e., power/control, self-defense, anger, intimidation, jealousy, poor communication, and retaliation) and very few gender-specific motives for perpetration of violence or intimidation emerged; however, the studies methodologies varied extensively suggesting the need for continued studies on IPV motivation and gender differences.

Women’s engagement in IPV is most often cited to be associated with their partners’ perpetration of aggression against them (Allen et al., 2009; Graves et al., 2005). Magdol et al. (1998) found that women who have experienced IPV were 13 times more likely to engage in violence toward their partner than non-victimized women. Studies have also shown that women who experienced psychological abuse and intimidation from their partners were 7 times more likely to perpetrate IPV than non-victims, suggesting a potential link between the trauma-related decompensation from emotional battering and fear to the use of violence among women (Kubiak et al., 2012; Leisring et al., 2003; Sullivan et al., 2005). However, psychological abuse, emotional neglect, intimidation, and physical violence are often co-occurring—further confounding conclusions regarding specific associations to women’s use of violence (Leisring et al., 2003). One could posit, a woman may have been controlled by a previous physically violent relationship and further controlled in the next relationship by threats and intimidation due to the trauma-related previous experiences of violence.

Additional studies suggest acts of violence and assault perpetrated by women are generally isolated events occurring within the context of people known and close to them (Durose et al., 2005; Kruttschnitt et al., 2002). A meta-analysis on female perpetrated physical violence reported that it is not uncommon and can result in the same degree of severity and injury as male perpetrated IPV (Archer, 2000; Carney et al., 2007; Leisring, 2011). Because of the relational nature of women’s use of violence, the research has predominantly focused on IPV, with a few studies addressing violence and aggression against non-intimate partners (Felson & Cares, 2005; Kubiak et al., 2017). One study found that past engagement in IPV increased the likelihood that women (by 4.4 times) would also be aggressive toward non-intimate partners, compared with women who did not engage in previous IPV, further indicating the need to explore women’s use of violence in general (Moffitt et al., 2001).

#### Childhood trauma and abuse

ACEs have been shown to increase the risk of women’s IPV both directly and indirectly (Allen et al., 2009; Kubiak et al., 2017; Dowd et al., 2005; Kernsmith, 2006; Rivera et al., 2014; Siegel & Williams, 2003; Sullivan et al., 2005). Kruttschnitt et al. (2002) found a direct correlation between childhood physical abuse and adult female perpetrated IPV. Pollock and Davis (2005) and Pollock et al. (2006) found that childhood victimization, substance use, and certain personality traits (e.g., repressed anger) each increased the likelihood of women’s use of violence against their partners. Studies have also demonstrated the mediating role of anger between sexual victimization, IPV, mental health, substance use disorders, and the perpetration of violence and intimidation among incarcerated women (Bonomi et al., 2006; Maneta et al., 2012). In addition, some literature has shown a stronger association between victimization and violence among women compared with men (Olatunji et al., 2010; Orth & Wieland, 2006).

#### Substance use and dependence

Additional literature indicates an indirect relationship, where substance use and mental health issues mediate the association between exposure to physical and/or sexual abuse and risk for criminal involvement (Kennedy et al., 2013; Tripodi & Pettus-Davis, 2013). White and Widom (2003) studied men and women with and without a history of childhood abuse and found that substance use and hostility (e.g., threats, intimidation, and temper) were mediators for the relationship between childhood adversity and IPV perpetration only for women. Another study including women in substance use treatment found bivariate relationships between a history of childhood adversity and adult-perpetrated violence against non-partners (Murray et al., 2008). However, these associations are not well understood and are often cyclical. Substance use creates a vicious cycle which increases risk of future assault and assault increases risk of substance use (Swan et al., 2005). Co-occurring substance use and mental health disorders have also been identified as correlates of women’s violence, particularly within criminal justice samples (Logan & Blackburn, 2009; Silver et al., 2008).

#### Criminal justice involvement

Childhood victimization has been previously linked to a pathway of criminal involvement (Brennan et al., 2012). These studies have shown that younger starting age of antisocial behaviors is significantly related to a trajectory of conduct problems, criminal justice involvement for girls, and adult-perpetrated violence (Broidy et al., 2003; Cote et al., 2002; Leban & Gibson, 2020; Messina & Grella, 2006; Moffitt et al., 2001). Additionally, a few studies have shown that 60% to 70% of women perpetrating IPV report a prior arrest (Babcock et al., 2003; Dowd et al., 2005). Others have not found significant differences in the likelihood of violence among incarcerated women with and without prior arrests (Kubiak et al., 2013).

Childhood victimization is a complex issue, often resulting in lifelong trauma and the development of harmful behaviors. Victimization becomes a pattern for women, occurring in childhood, adolescence, repeated from relationship to relationship, and then as a contributing factor to the use of aggression towards partners and others, validating the need for further research on recovery needs and appropriate program development (Bloom et al., 2003). Recent pilot research within the California Department of Corrections and Rehabilitation (CDCR) has found that a trauma-specific brief intervention (i.e., *Healing Trauma: A 6-session Brief Intervention for Women*—Covington & Russo, 2016) and a 20-session intensive violence prevention programs (i.e., *Beyond Violence*—Covington, 2015) have shown significant positive results decreasing anger, aggression, and hostility, and increasing mental health well-being and emotional regulation (Messina & Calhoun, 2021; Messina & Zwart, 2021; Messina et al., 2020).

### Pathways perspective

A pathways perspective recognizes the specific challenges and realities in women's lives and recognizes that men and women have different pathways to criminal activity and substance use (Blanchette & Brown, 2006; Chitsabesan & Bailey, 2006; Daly, 1992; Gavazzi et al., 2006; Gehring, 2018; Reisig et al., 2006; Salisbury & Van Voorhis, 2009; Wattanaporn & Holtfreter, 2014; Wright et al., 2012). Brennan et al. (2012) identified eight reliable, yet complex, pathways to women’s recidivism linking multiple women-centered factors to previous literature, including sexual and physical abuse; lower social capital; poor relational functioning; and extreme mental health issues. Pathways’ theorists have also linked ACEs to women’s violence as contributing factors to the trajectory of the “harmed becoming the harming” and as primary predictors of onset of criminal activity for women (Benda, 2005; Bloom, 1996; Bloom et al., 2003; Daly, 1992; Maneta et al., 2012; Messina & Grella, 2006; Owen et al., 2017).

Messina and Grella (2006) assessed childhood victimization and household dysfunction among 500 women on parole and found the cumulative number of ACEs had a strong and graded relationship with earlier engagement in criminal activity and substance use, as early as 14 years old. ACEs were also significantly correlated with adolescent pregnancy, homelessness, and prostitution. Employing Cox proportional hazards models, Benda (2005) showed that childhood and recent abuses, urban residence, living with a criminal partner, selling drugs, anxiety, depression, fearfulness, and suicidal thoughts were stronger predictors of recidivism for women than for men. Other studies contend that women-centered factors, such as financial dependence, and how they intersect with race/ethnicity are a more accurate depiction of criminal involvement and recidivism among women (Boppre, 2019; Huebner et al., 2010). Hamilton et al. (2017) found that predictive factors of recidivism for 8815 women were primarily related to social support (e.g., minor children, no child support, legal contact restrictions) and victim/offender characteristic prevalent among women (e.g., IPV and prostitution).

In sum, research focused on justice-involved women has begun to outline the role of ACEs—which includes childhood exposure to criminality, addiction, out-of-home placement, and incarceration of a parent, in the transition toward high-risk behaviors (Grella et al., 2005; Messina & Grella, 2006; Minh et al., 2013). Such traumatic events could also include early exposure to the criminal justice system, a factor that has not previously been examined as a correlate of women’s use of violence. Additionally, as there is an intergenerational transmission of victimization and violence, it is vital that services are oriented to the needs of women and their families to stop the cycle of victimization and dysfunctional relationships (Black et al., 2010).

### Current study

This study draws upon the pathway’s framework to explore gaps in knowledge on women’s perpetration of violence by examining childhood victimization and early criminal justice involvement, while adjusting for previously found correlates of violence (i.e., substance use and long-term criminal justice involvement). Specifically, this study examines the cumulative impact of childhood victimization, age of first arrest, and number of arrests over one’s lifetime as main risk factors associated with women’s perpetration of violence as an adult. While prior research is limited in differentiating between forms of women’s violence, this study further examines the impact of these factors on various forms of adult perpetrated violence (intimidation, and physical violence). Moreover, this study examines violence directed at either an intimate partner or others.

Hypotheses: Based on the previous literature and pathways framework, the following hypotheses were examined:

#### Hypothesis 1

The cumulative experience of childhood victimization (under age 18) will be associated with significantly higher scores on adult perpetration outcomes (i.e., intimidation and physical violence).

#### Hypothesis 2

Early involvement in the criminal justice system (arrest before age 18) will be associated with significantly higher scores on adult perpetration outcomes.

#### Hypothesis 3

Long-term criminal justice involvement (greater number of lifetime arrests) will be associated with significantly higher scores on adult perpetration outcomes.

#### Hypothesis 4

Poly-substance use 12 months prior to incarceration will be associated with significantly higher scores on adult perpetration outcomes.

## Methodology

### Participants

This study is a secondary analysis of data collected from 1118 participants in the trauma-specific program “Healing Trauma: A Brief Intervention for Women” (Covington & Russo, rev 2016) in two California prisons. Table 1 shows the participant characteristics and self-report histories by prison (pilot study samples 1 and 2) and level of custody (pilot study sample 3). Although the security housing unit (SHU) population is from the same facility, participants are shown as a separate group due to the severity and frequency of violence of this population of women. For the total sample of 1118 participants, Hispanic/Latina and White were the largest racial groups represented (33% and 28% respectively) followed by African American/Black (19%). The mean age was 37 years with 92% of the participants reporting an education level of less than High school/GED prior to incarceration,^1^ and the majority reporting being single/never married (45%). Additionally, 35% of participants indicated age of first arrest being younger than 18, with a mean number of lifetime arrests of 13.5 (*SD* = 21.3) and mean years incarcerated of 7.1 (*SD* = 7.4). Sixty-two percent were incarcerated for a violent or serious offense(e.g., murder, manslaughter, attempted murder, assault, or robbery and burglary). Participants also reported using two or more substances (2.2., *SD* = *1.6*) regularly during the 12 months prior to incarceration. On average, the participants report over three different forms of childhood victimization (SD = 3.3) before the age of 18.

**Table 1:** Participant characteristics by Prison

	Prison I *n* = 256 %/mean(*SD*)	Prison II *n* = 804 %/mean(*SD*)	SHU *n* = 58 %/mean(*SD*)	Total* N = 1118 %/mean(*SD*)	Sig *p* < .05
*Perpetrated violence*					
Intimidation (sum) (0–14)	2.2 (2.8)	1.4 (2.4)	2.1 (2.7)	1.6 (2.5)	*
Physical violence (sum) (0–13)	2.8 (3.0)	2.2 (2.6)	3.6 (3.1)	2.4 (2.8)	*
Types of abuse experienced (count) < 18	3.6 (3.6)	2.8 (3.1)	4.3(3.4)	3.1 (3.3)	*
*Criminal justice predictors*					
Age of first arrest < 18	39.6	31.7	60.7	35.0	*
Number of arrests	11.0 (19.5)	14.2 (21.9)	14.6 (19.4)	13.5 (21.3)	
*Substance use predictors*					
Number of substances used (0–8)	2.0 (1.4)	2.2 (1.6)	2.8 (1.9)	2.2 (1.6)	*
*Race/ethnicity*					*
White	25.8	30.3	13.8	28.4	
Hispanic/Latina	28.1	34.3	36.2	33.0	
Black/African American	24.6	16.2	27.6	18.7	
Multiracial	12.1	11.2	22.4	12.0	
Other/unknown	9.4	8.0	0.0	7.9	
*Age (at baseline interview)*	40.1 (12.5)	36.0 (9.9)	33.5 (8.8)	36.8 (10.6)	*
*Education (HS/GED or higher)*	4.5	5.1	6.9	5.1	
*Marital status*					*
Single/never married	41.7	45.1	64.9	45.4	
Living together/legally married	30.7	30.0	21.1	29.7	
Separated/divorced/widowed	27.6	24.8	14.0	24.9	

^*^N’s vary for each variable due to missing data. Arrest variables had the highest amount of missing data with a total of 1076 valid cases for number of arrests, and 1091 valid cases for age of first arrest

### Procedures

The original data for the pilot studies were collected from 2017 to 2019 and the evaluation approvals were obtained by the UCLA Institutional Review Board and the Office for Protection of Research Subjects.

#### Pilot study recruitment

*Healing Trauma* Program Coordinators posted flyers about the program in the facilities housing “high risk and high need” women. Women volunteered to participate in the intervention and evaluation. Research staff provided self-administered pre- and post-surveys at each facility prior to the program entry. On average, the surveys were completed within 45 min. In addition to demographic questions, and criminal justice and substance use histories, the baseline instrument included scales on perpetration and victimization. Due to restrictions of the pilot study funding, women were not compensated for participation.

#### Pilot study program

*Healing Trauma* is a 6-session psychoeducational trauma curriculum designed for women who have experienced trauma associated with ACEs (Covington & Russo, 2012, rev 2016). The participants were housed in multiple levels of secure detention, including the “high risk—high need” housing (i.e., housing for women with multiple disciplinary infractions), the reception center (i.e., housing upon prison entry for risk classification), and the SHU (segregation for violent acts toward others or custody officers). The program was delivered twice weekly, with 2.5-h sessions, over six weeks. Groups were closed and included 8–10 women. The series of pilot studies from over 1118 incarcerated women in California showed that the 6-session brief intervention was significantly impactful revealing reductions in anger, aggression, hostility, current traumatic distress, anxiety and depression (Messina & Zwart, 2021; Messina et al., 2020). Moreover, the greater number of ACEs reported among the women increased the likelihood of program gain on all mental health and aggression outcomes (Messina & Schepps, 2021; Sigler et al., 2020).

### Measures

The dependent variables in the current study included multiple forms of adult perpetrated intimidation, and physical violence against an intimate partner or someone other than an intimate partner.

#### Perpetration of physical violence or intimidation

The dependent variables were measured through a modified index of perpetration history based on several of the items from the Conflict Tactics Scales (Straus, 1979; Straus et al., 1996) and the Abuse Behavior Inventory (Shepard & Campbell, 1992). The 7-item intimidation scale included items such as, “threatened to kill,” “threatened to harm family or friends,” and “threatened to take children.” Additionally, the physical violence scale contained 7 items such as, “pushed, grabbed, thrown”, “slapped, kicked, punched”, “restrained physically”, and “stabbed,”.” The items were asked in relation to whether the women perpetrated each item toward a romantic partner and then again in relation to whether they perpetrated each item against someone else as an adult, respectively (see Table 2). Responses altered between *no* (0) or *yes* (1) and were summed to indicate the total number of items endorsed for each subscale. If a respondent indicated that they had perpetrated violence against a romantic partner and against another adult, both were included in the sum. The summed scores of yes and no yielded a Cronbach’s alpha of 0.860 (*n* = 1118) for intimidation, and 0.840 (*n* = 1118) for physical violence.

**Table 2:** Perpetration of violence and victimization items (*N*=1,118)

*Perpetrated violence*	Intimate partner %	Other than intimate partner %	Composite score sum > 0 %
*Intimidation*	32.0	31.0	40.8
Threatened to kill	14.0	15.5	
Threatened to hurt	25.2	25.4	
Threatened with a gun, knife, weapon	14.7	18.0	
Threatened to harm family members or friends	6.1	11.3	
Threatened to take custody of the children	5.0	2.4	
Threatened to kill yourself to intimidate	9.8	4.1	
Threatened to have hospitalized	2.7	4.1	
*Physical Abuse*	49.0	44.6	57.3
Pushed, grabbed, shoved, thrown	41.9	36.9	
Held to keep from leaving or restrained physically	37.5	10.5	
Slapped, punched, kicked	42.0	38.0	
Burned	1.7	2.3	
Beat unconscious	1.7	3.1	
Choked	10.9	8.1	
Shot, stabbed, cut, or used a gun, knife, weapon against	11.0	16.2	
*Types of Abuse Experienced*	***< 18 years*** *%*		67.3
Ever been pushed	53.5		
Ever been hit, slapped,	52.8		
Ever been kept from leaving or restrained physically	29.8		
Ever been burned	8.8		
Ever been beaten	7.2		
Ever been choked	16.5		
Ever been shot	13.1		
Ever been threatened to be killed	15.7		
Ever been threatened to be hurt	30.5		
Ever been threatened with a gun	15.0		
Ever had someone threaten to harm family members	14.5		
Ever had someone threaten to take custody of children	5.9		
Ever been threatened to have you hospitalized	7.7		
Ever been forced into an unwanted sexual act	34.7		

Independent variables also used the Conflict Tactics Scales and Abuse Behavior Inventory; however, the items were based on responses to specific questions regarding victimization under the age of 18, age of first arrest under the age of 18, number of lifetime arrests, and poly-substance use 12-months prior to incarceration. Demographic factors used as covariates included race, education, and age, based on prior practice in prison studies on substance use and psychological outcomes for women (e.g., Robbins et al., 2009; Sacks et al., 2012,).

#### Victimization prior to the age of 18

Abuse history was measured through items where respondents were asked whether they had experienced various forms of violence including being forced into an unwanted sexual act, intimidated, and physical abuse prior to the age of 18. Responses of ‘yes/no’ were summed to indicate the total number of items endorsed for the predictor of the cumulative experience of abuse under the age of 18. On average, women reported 3.1 (SD = 3.3) different forms of abuse prior to the age of 18.

#### Criminal activity

Age of first arrest was self-reported in the pre-program questionnaire with a response to “How old were you the first time you were arrested?” Responses were coded to a dichotomous variable with those arrested at an age younger than 18 and those arrested at 18 or older. The number of arrests was based on the question, “How many times during your lifetime have you been arrested?”.

#### Substance use history

Number and type of substances used were reported in the pre-program questionnaire with a response to “Did you drink alcohol or use drugs during the 12-months prior to your arrest?” A follow-up question asked respondents to select the type of substance used (i.e. alcohol, marijuana, cocaine/crack, heroin/opiates, amphetamines/methamphetamine, prescription drugs, designer drugs (ecstasy, MDMA), hallucinogens). The sum of these selections was used to assess poly substance use.

#### Data analyses

Preliminary analyses included bivariate comparisons for dependent variables and correlates by facility (i.e., Chi-Square and mean comparisons using ANOVA) to examine baseline differences in the pooled sample sub-groups. Generalized Estimating Equations regression models were used to examine the relationship between the predictors and the number of instances reported for perpetrated intimidation and physical violence measured as non-negative count variables. Over-dispersion of the dependent variables (intimidation *mean* = 1.58, *var* = 6.42; physical violence *mean* = 2.4, *var* = 7.64) necessitated the use of the negative binomial link function and incidence rate ratios (IRRs) are used to report effects. Additionally, observations were nested within facility to account for differences between the participants from the two sites on victimization, criminal justice, and substance use backgrounds. Clustering or nesting in the regression model specifies how to estimate the variance co-variance matrix where the standard errors allow for within group correlations and assumes independence between the sites. After listwise deletion^2^ the regression models consisted of a sub-sample with 1048 women for the two dependent variables, respectively with missing data for 70 women largely stemming from arrest-related variables. Race/ethnicity categories were dichotomized, and the Other/Unknown category combines Asian, Pacific Islander, American Indian and missing data. Based on group size as well as historical practice of comparing the predominant group to historically marginalized, the reference group was ‘white’ for race/ethnicity and ‘less than HS/GED’ for education level.

## Results

Table 2 shows the distribution for adult perpetration of violence and victimization in childhood for individual items used in the composite scores. Prior to the age of 18*,* 67% of the women reported that they had been victims of physical abuse, intimidation or sexual abuse. With regard to perpetrated violence, 32% reported perpetrating intimidation against an intimate partner, and 31% reported perpetrating it against someone other than an intimate partner. For physical violence, 49% reported perpetrating it against an intimate partner, and 45% reported perpetrating it against someone other than an intimate partner. Since percentages were similar for perpetration of violence against an intimate partner and against someone other than an intimate partner, these were combined in the regression models.

Findings from the regression analyses for each model predicting the  two types of perpetrated violence are presented in Table 3, effects are reported as incidence rate ratios (IRR).^3^ Model fit statistic was derived from a Baysian Information Criteria (BIC) (Schwarz, 1978) test, which is one method of overcoming issues of calculating Wald Chi-Square tests where the number of clusters (i.e., pilot prison samples 1, 2, and 3) is substantially less than the number of parameters (i.e., 14). Relative to this study, a BIC difference (ΔBIC) of greater than ten shows evidence favoring the full model vs. the null model is strong. In all three models, the model selection technique favored the full model over the null model (intimidation BIC difference = 132.62; and physical violence BIC difference = 161.43).

**Table 3:** Regressions predicting Intimidation and Physical Violence

	Intimidation Model I (N = 1048)		Severe physical violence Model II (N = 1048)	
	*IRR*	CI: 95%	*IRR*	CI: 95%
Number of abuse experiences < 18	1.17**	1.15–1.19	1.13**	1.12–1.13
Age of first arrest < 18	1.38**	1.30–1.47	1.26**	1.13–1.40
Number of arrests	1.00**	1.00–1.01	1.00*	1.00–1.01
Number of substances used	1.22**	1.17–1.27	1.14**	1.13–1.14
Black/African American	1.17	0.79–1.76	0.97	0.81–1.17
Hispanic/Latina	1.06	0.89–1.27	1.06	0.97–1.15
Multiracial	0.99	0.89–1.08	1.14	0.94–1.39
Other/unknown	1.52**	1.32–1.76	1.40**	1.24–1.57
Age	0.99	0.99–1.00	0.99	0.99–1.00
Education (HS/GED or higher)	0.93	0.59–1.47	0.92	0.62–1.36

Reference Group: White, less than HS/GED

^*^p < .01; **p < .001

As hypothesized, the predictors of adult-perpetrated violence (i.e., childhood victimization, early and ongoing criminal involvement, and adult substance use) were significantly related to all forms of women’s use of violence. The strongest significant predictor of adult perpetration of violence and intimidation was early involvement in the criminal justice system (i.e., first arrest under the age of 18) for all three outcomes: intimidation (IRR = 1.4; *p* < 0.01, 95% CI [1.30, 1.47], and physical violence (IRR = 1.3; *p* < 0.01, 95% CI [1.13, 1.40]. With a positive association in all three models, reported age of first arrest as a minor corresponded to a greater number of types of violence and intimidation perpetrated against others and romantic partners.

The number of types of abuse experienced under the age of 18 was the second strongest and significant predictor of adult perpetration of violence and intimidation for all three outcomes: intimidation (IRR = 1.2; *p* < 0.01, 95% CI [1.15, 1.19], and physical violence (IRR = 1.1; *p* < 0.01, 95% CI [1.12, 1.13]. Similar to age of first arrest as a minor, the cumulative number of forms of childhood victimization was associated with a greater number of types of violence and intimidation perpetrated for each outcome, respectively.

Also, as self-reported number of substances used 12-months prior to incarceration increased (poly drug use), the number of types of adult perpetration of all three outcomes also increased: intimidation (IRR = 1.2; *p* < 0.01, 95% CI [1.17, 1.27], and physical violence (IRR = 1.1; *p* < 0.01, 95% CI [1.13, 1.14]. The total number of arrests also shared a positive association with each of the three outcome measures: intimidation (IRR = 1.0; *p* < 0.01, 95% CI [1.00, 1.01], and physical violence (IRR = 1.0; *p* < 0.01, 95% CI [1.00, 1.01]. Lastly, there was a significant association with race/ethnicity and all outcomes, whereby women who identified as Asian, Pacific Islander, American Indian (and unknown) shared a positive association with the perpetration of violence outcomes compared to women who identified as White.

## Discussion

Drawing from the pathway’s framework, this study examined experiences from childhood, adolescence, and adulthood as potential correlates of women’s perpetration of violence and intimidation against romantic partners and others. All hypotheses were supported, with first arrest under the age of 18 as the strongest predictor of all forms of adult-perpetrated violence, followed by the cumulative number of childhood victimization, and poly-substance use prior to incarceration. These factors were similarly associated with the respondent’s perpetration of violence against either an intimate partner *or* others. Previous literature has shown that ACEs have a strong and graded relationship with earlier involvement in substance use and criminal activity (Grella et al., 2013; Fazel et al., 2006; Messina & Grella, 2006; Saxena et al., 2016; Tripodi & Pettus-Davis, 2013; Tusher & Cook, 2010)—the strong impact of early age of criminal involvement may serve as a mediating risk factor influencing women’s ongoing harmful behaviors, dysfunctional relationships, threats and intimidation, and escalation to violence.

There was also a significant and positive association for those who reported “other/unknown” race/ethnicity for perpetration of violence and intimidation. This small group of women who identified as Asian, Pacific Islander, American Indian, or “unknown” had higher mean outcomes for the dependent variables at the bivariate level in comparison to the reference group in the regression models (i.e., White), which remained regardless of added variables in the models. It is difficult to make inferences regarding this finding given the small number of women overall (8%) in this category. Given the literature outlining racial disparities of incarcerated populations, this finding requires further investigation.

Experiences of childhood victimization, the connection to earlier and ongoing criminal involvement, and subsequent acts of perpetration are cumulatively and inextricably linked. The finding that childhood victimization and arrest experienced as a minor are predictors of perpetration of adult violence underscores the need for early assessment and appropriate interventions. However, thousands of women currently incarcerated for violent offenses are in immediate need of services to manage and lesson their trauma-related harmful behaviors.

### Strengths

A primary strength of this study was the large sample size with  extensive interview data, allowing the examination of specific data elements to explore the trajectories of violence in the women’s lives, as victims and as perpetrators. Previous literature has outlined the impact of adult mental health problems and substance use histories as primary factors predicting women’s use of IPV. This study builds upon that body of literature by exploring the impact of cumulative childhood experiences of abuse and early involvement with the criminal justice system (also a traumatic event) as risk factors for women’s use of IPV and violence against non-intimate partners. The results from the modified perpetration and victimization histories survey further demonstrated the high prevalence of childhood victimization and the high prevalence of adult perpetration of physical violence and intimidation among the pooled sample of women incarcerated for violent crimes, enhancing the reliability and the validity of the findings.

### Limitations

The generalization of the findings may be limited to incarcerated women in California, as the purpose of the original studies was to provide a trauma-specific violence prevention program to women convicted of violent crimes. Another purpose was to explore correlates of women’s use of violence; thus, the sample provided a good fit for the hypothesized models in this secondary data analysis. The variance explained in the model was relatively small, suggesting the influence of other potential factors that are not accounted for in these analyses. It is possible that structural contexts (e.g., socioeconomic factors) mediated or mitigated outcomes. Also, the study findings rely solely on retrospective self-reported experiences and behaviors, limiting the longitudinal implications of the cumulative and life-time impact of adverse experiences on women’s use of violence.

### Policy implications

Expanding the understanding of the trajectory of victimization and violence in women’s lives provides implications for interventions that address the resulting trauma of these events (e.g., the *Healing Trauma Program*), both during custody and after release. Although it is crucial to explore all factors associated with male and female patterns of violence and aggression, it is clear, that ACEs have been shown to increase the risk of women’s trajectories of violence. The traumas suffered in childhood are often re-occurring and escalating throughout the lives of justice-involved women and can further impact their recovery. Providing trauma-specific violence prevention treatment for women prior to release and within the community could help to negate the cycle of violence in their lives.

A growing body of literature has shown that trauma-specific and violence prevention treatment for incarcerated women can increase psychological well-being, and decrease violence and aggression, further creating safer custody environments (Kubiak et al., 2016; Messina et al., 2010; Messina et al., 2014; Messina & Calhoun, 2021; Messina & Zwart, 2021; Messina et al., 2020; Saxena et al., 2014).^4^ Additionally, tools that incorporate and accurately assess the experience of trauma are vital to guide the development and delivery of early trauma-specific interventions for girls involved in the criminal justice system.^5^ The past and current findings further support rethinking policies and procedures surrounding the culture of custody environments, and the movement toward trauma-informed care in corrections organizations.

## Conclusion

There is potential for appropriate preventative measures to mitigate the trajectory of victimization to violence among women and girls who have experienced ACEs. Particularly the effect of age suggests the need for a trauma component to youth-offender programs that address associated problems such as conduct disorder, continued involvement in criminal activity, and other harmful behaviors (e.g., substance use or risky sexual behaviors). It is also important that trauma-informed coping strategies, interpersonal communication and problem-solving skills, be developed at an early age. Trauma-specific interventions for incarcerated women can also be feasibly implemented and expanded to address these early childhood experiences to heal the trauma from the past (Messina & Calhoun, 2021).

It is clear from the analyses that childhood victimization, earlier criminal involvement, and habitual criminal activity in women’s lives have a role in the pathway to perpetration of violence. Yet there is much to untangle given the complex realities of justice-involved women’s lives. Future research should continue the exploration of the trajectories of violence in women’s lives using retrospective and prospective studies to explore other predictive factors and the complex interaction of childhood and ongoing violence and victimization.

## Data Availability

This data belongs to the California Department of Corrections and Rehabilitation and is only available with their permission. The materials used can be provided by the authors.

## Footnotes

1 For current highest level of education, 703 participants (56%) reported a HS/GED or higher, however 88% of this group obtained their GED/HS Diplomas in prison leaving a large majority without a HS/GED prior to prison.

2 In the listwise deletion technique, if a case is missing data on one of the variables in the specified multivariate statistical procedure, it is eliminated from the analysis sample.

3 If the odds/incidence rate ratio is reported to be < 1 it is evidence for a negative association, and if the odds ratio is reported as > 1 then there is evidence for a positive relationship (Holcomb et al., 2001).

4 See Covington, S. (2015). *Beyond violence: A prevention program for criminal justice-involved women participant workbook*. John Wiley & Sons. See Covington, S, & Russo, E. (2012, rev. 2016). *Healing trauma: A brief intervention for women.* Center City, MN: Hazelden Publishing. www.stephaniecovington.com.

5 See Covington, S., Covington, K., & Covington, M. (2004, rev. 2017). *Voices: A program of self-discovery and empowerment for girls,* 2nd Edition. Carson City, NV: Change Companies.
